# Carbamazepine-induced Stevens-Johnson syndrome progressing to toxic epidermal necrolysis: a rare clinical image

**DOI:** 10.11604/pamj.2024.47.44.42577

**Published:** 2024-02-06

**Authors:** Ramdinmawii Ralte, Deeplata Mendhe

**Affiliations:** 1 Department of Community Health Nursing, Smt. Radhikabai Meghe Memorial College of Nursing, Datta Meghe Institute of Higher Education and Research, Sawangi (Meghe), Wardha, Maharashtra, India

**Keywords:** Stevens-Johnson syndrome, induced, carbamazepine

## Image in medicine

Stevens-Johnson syndrome is a rare and severe medical illness affecting the skin and mucous membranes. It is frequently caused by an adverse reaction to a medication; however, infections can also be the cause. It is characterized by a skin rash that begins with flu-like symptoms and progresses to the formation of a painful, red, or purplish rash that spreads and blisters. It presents a variety of symptoms, including fever, exhaustion, and general malaise. Here we present a case of a 35-year-old woman admitted to the hospital with complaints of skin rash, pain, and blisters all over her body for 1 week. The patient has been taking carbamazepine for 1 month due to her epileptic episode, after which the skin rash and blisters started to develop, and feel pain. The rash started on her face and spread to the upper limbs and all over her body. She has been receiving a tablet of methylprednisolone 4mg thrice a day, an injection of Tazar 4.5gm thrice a day intravenously, an injection of tramadol 50mg with normal saline infusion, a tablet of Montelukast 10 mg once a day at night time, fusidic acidic ointment. The rash and skin peel off resembling severe burns progressing to toxic epidermal necrolysis.

**Figure 1:**
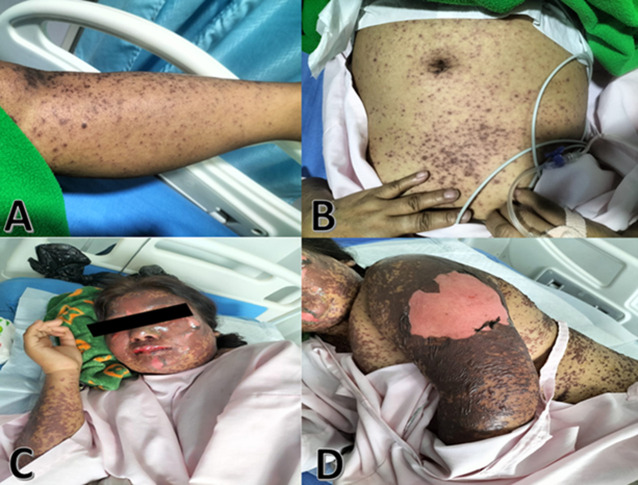
A) skin rash and pustules with blisters on the skin of the face; B) rash of small purplish spots on the abdomen; C) blister ruptured and skin peeling on the face; D) skin peeling in sheets on the arm

